# Monthly Summary

**Published:** 1873-01

**Authors:** 


### MONTHLY SUMMARY.

*A Curious Gum Varnish.*—It is well known that the ordinary gum arabic so freely used in photography consists chiefly of the calcium salt of a peculiar acid called "gummic acid" The other salts of the acid are but little known, though the acid itself can be isolated by a process which we shall give presently. Our object in referring to this matter now, however, is to draw attention to a body which appears to be an iron salt, and to possess properties likely to render it of some value.

If we take a solution of gum, and add to it an acid solution of perchloride of iron, no change takes place when the mixture is not exposed to strong light; but if instead of strongly acid solution, we take one which contains as little free acid as possible, and add to our mucilage gum, the latter becomes a complete jelly, stiff and strong, immediately on mixture of the two liquids. This jelly has a reddish brown color, and dries up to an even horny layer, very different from gum in appearance. We have coated paper with this substance as follows:—A solution of the liquor of perchloride of iron of the British Pharmacopœia was taken, and to it ammonia cautiously added with agitation until a permanent precipitate made its appearance. The liquor was then filtered, paper saturated with the solution, and allowed to dry in the dark. The coated sheets were then floated on some thick mucilage of gum arabic. The surface of the paper was thus covered with an even layer of the "gummate of iron."

When the paper carrying the iron is first coated with the mucilage, the color does not at once change, but presently a strong yellowish brown tint is produced, and the gum "sets," and then the layer dries up, leaving the paper very flexible for a long time and highly glazed. When paper so treated is allowed to stand in cold water, a certain amount of the gum dissolves, but a considerable quantity is retained by the iron. The portion but little affected by cold water is, however, easily removed by the hot solvent, some iron at the same time passing into the solution. When the gummate of iron paper is exposed to light for some time, the gum is less easily dissolved by hot water, and less affected by cold water, than that which has not been so treated. The paper is distinctly sensitive to light, as most other iron-prepared papers are. If washed in water containing a small quantity of ammonia, the brown tint of the paper is increased, and the gum is somewhat less easily dissolved out by water, though by treatment with a little very dilute acid, the gum and much of the iron can be dissolved out.

The gummic acid above referred to can be prepared by precipitating a solution of gum arabic with acetate of lead, washing the precipitate, and then suspending it in water through which a current of sulphureted hydrogen is passed. Sulphide of lead is formed and gummic acid set free. The latter can then be obtained on evaporating the solution.—*British Journal of Photography.*

---

*Neuralgia Cured by Shock.*—The patient, aged 58, was attacked in the autumn of 1858 with violent neuralgic pains in the right arm and shoulder. There had been no injury to the joint, and its motion was unimpaired. All the nerves of the arm and forearm were affected. The pain at times extended to the spine and more rarely to the left shoulder. Treatment had but little effect. The patient's general condition was deteriorating and the arm was losing power and size. On the 23d of January, 1859, after an evening of great suffering, he slipped and fell on the ice, striking the affected shoulder with great violence. The pain was intense, and he thought at the time that the "arm was torn from the socket." The pain soon ceased, and was followed by a feeling of warmth and "naturalness." The neuralgia ceased and has not yet returned—more than thirteen years since the accident.

Dr. Rand puts the case on record as a simple matter of fact, without attempting an explanation.—*Med. Times.*

---

*Mixed Vapors for Anæsthesia.*—Dr. J. D. Davis (*Medical Record,*) in commenting upon this subject remarks that the mixture recommended by the Committee on Anæsthetics, of the American Medical Association, deserves to be more universally known and used, its advantages being—the small quantity required; the rapidity of its action; the shortness of the stage of excitement (it often being wanting entirely;) the absence of distressing after effects; its comparative safety over chloroform; being compounded in definite proportions, it can be scientifically administered; the pungent, stifling odor of the ether and the burning sensation of the chloroform, are to a great extent overcome; the sedative effects of chloroform are modified by the exciting influence of ether, while to both is added the stimulant alcohol. The mixture is composed of alcohol, one part; chloroform, two parts; and ether, three parts. It is easily remembered by taking the initials in the order of the alphabet: A., C., E., the corresponding number of parts being 1, 2, 3.—*Med. Examiner.*

*External Use of Fuming Nitric Acid.*—Professor Patruban (*Memorabilien*, June, 1872,) says that this heroic caustic is indicated on account of its rapidity and drying-up of the tissues. It stops all effusion of blood, and leaves good scars. He used it for warts and other protruberances of the skin, as condylomata, epulis, hemorrhoids, and tumors of the nature of nævus.—*The Doctor*.

---

*Dentistry Extraordinary.*—The following case is reported in the *Medical Times and Gazette*:—A surgeon was charged with having pulled out a boy's tooth against his will. He pleaded guilty. It was explained that a number of boys had annoyed the doctor, and that he seized one of them, took him into his house, and extracted one of his front teeth against his will. The sheriff fined the accused one pound or seven days in prison. The fine was paid.

---

*Cement for Attaching India Rubber to Wood or Metals.*—A cement that will form a firm union of rubber with either wood or metals consists of a solution of shellac or gum lac in ammonia. Finely pulverized lac is dissolved in about ten times its weight of concentrated ammonia. The solution takes place in the course of a few days without the application of heat, or sooner if slightly warmed.—*Druggists' Circular*.

---

*Test for Creasote.*—It is said that creasote may be distinguished from carbolic acid (which is sometimes substituted for it,) by mixing the suspected sample with pure glycerine; pure creasote is insoluble in this medium, whereas carbolic acid makes a bright solution, and when present in considerable quantity it makes creasote also soluble in the mixture.—*Druggists' Circular*.

---

*Ready Mode of Preventing Infection.*—There is perhaps no plan of preventing infection so ready as the production of sulphurous acid by the combustion of sulphur. To disinfect a bed, whilst the patient is temporarily removed from it, pass a copper warming pan into the bed, containing a few live embers and a little sulphur. The pan should be moved about during the ignition of the sulphur. By burning sulphur in an open vessel, closets, carriages, passages, and vacated chambers of the sick may be easily disinfected. Clothing may be lightly sponged over or sprinkled with water containing a little well mingled sulphur, and then ironed with a flat iron heated to a temperature which will cause volatilization of the sulphur without burning the linen.—*Mr. J. Sartin*, in *Braithwaite*.

